# Expressions of Transforming Growth Factor β_1_ Signaling Cytokines in Aortic Dissection

**DOI:** 10.21470/1678-9741-2018-0129

**Published:** 2018

**Authors:** Shi-Min Yuan, Hong Lin

**Affiliations:** 1 The First Municipal Hospital of Putian, Teaching Hospital, Fujian Medical University, Putian, Fujian Province, People's Republic of China.

**Keywords:** Aneurysm, Dissecting, Coronary Artery Disease, Intercellular Signaling Peptides and Proteins, Blotting, Western

## Abstract

**Objective:**

To demonstrate the underlying mechanisms of aortic dissection compared to those of coronary artery disease in terms of the transforming growth factor-beta (TGF-β) signaling pathway.

**Methods:**

Twenty consecutive aortic dissection patients and 20 consecutive coronary artery disease patients undergoing a surgical treatment in this hospital were enrolled into this study. The aortic tissues were sampled and the TGF-β_1_ and its receptor TGF-β receptor I (TβRI) were detected by Western blotting assay.

**Results:**

TGF-β_1_ and TβRI were positively expressed in the aortic tissues in both groups by Western blotting assay. The expressions of the two proteins were significantly higher in the aortic tissue of patients with aortic dissection than in those with coronary artery disease. The quantitative analyses of the relative gray scales of the proteins disclosed close correlations between the expressions of TGF-β1 and TβRI in both the study and control group patients.

**Conclusions:**

The aortic remodeling of aortic dissection might differ from that of coronary artery atherosclerosis concerning the nature, mechanism, mode, and activities of TGF-β signaling pathway. The development of aortic dissection could be associated with a significantly enhanced function of TGF-β_1_/Smad signaling transduction as a result of aortic remodeling incorporating both vascular injury and repair.

**Table:** 

Abbreviations, acronyms & symbols	
**AD**	**= Aortic dissection**
**Akt**	**= Protein kinase B**
**CAD**	**= Coronary artery disease**
**EMT**	**= Epithelial-to-mesenchymal transition**
**MAPK**	**= Mitogen-activated protein kinase**
**mRNA**	**= Messenger ribonucleic acid**
**NmuMG**	**= Normal murine mammary gland**
**PI3K**	**= Phosphatidylinositol-3-kinase**
**RNA**	**= Ribonucleic acid**
**TGF**	**= Transforming growth factor**
**TGF-β**	**= Transforming growth factor-beta**
**TβRI**	**= Transforming growth factor-beta receptor I**
**TβRII**	**= Transforming growth factor-beta receptor II**

## INTRODUCTION

Transforming growth factor-beta (TGF-β) is a multi-functional cytokine, which belongs to the TGF superfamily, signaling through transmembrane serine/threonine kinase receptors and Smad proteins. It is usually in a non-activated state and becomes activated when binding to the cell surface receptor, initiating the intracellular signaling transduction. TGF-β plays its biological roles via TGF-β receptors I and II (TβRI and TβRII) on the membranous surface. TGF-β stimulates the production of matrix proteins in vascular smooth muscle cells and matrix protein synthesis, leading to vascular remodeling^[1]^, responsible for aortic aneurysmal formation and possibly coronary artery disorders^[2]^.

Aortic dissection (AD) is the most dangerous cardiovascular disease and it is associated with an extremely high mortality if left without timely diagnosis and treatment^[3]^. Marfan syndrome is a typical genetic defect with significant propensity for development of AD^[4]^. It has been noted that endothelial dysfunction of the aortic wall in Marfan patients is caused by *fibrillin 1* gene mutations, thus leading to aortic wall fragility and predisposing to AD^[5]^. In Marfan syndrome, the microfibrillar deficiency would cause inadequate matrix sequestration, with subsequent activation of TGF-β^[6]^. The increased level of TGF-β is associated with an upregulation of proteases, such as matrix metalloproteinases, and they are responsible for the remodeling process of the aortic wall extracellular matrix^[7]^.

TGF-β is an important cytokine involved in the developmental process of heart and vessels^[8]^. It is upregulated in relation to injury of vascular walls as a mediator of the fibrotic response, and therefore the upregulation of TGF-β is a result other than a cause of vascular injury^[9]^. An excessive expression of TGF-β may also hint the progression of secondary disorders^[10]^. The plasma TGF-β has obtained noteworthy attention in recent years as a reliable biomarker for the evaluation of therapeutic effects of aortic remodeling in Marfan patients^[5]^. However, the mechanisms that TGF-β signaling regulates during the developmental process of AD and whether TGF-β is increased in aortic conditions in non-Marfan adults remain unknown. Moreover, the evaluation of cytokine expression of TGF-β_1_ signaling pathways by Western Blotting assays has not been sufficiently described. In order to determine the roles of TGF-β as a potential biomarker of AD development, a prospective study was designed to assess the expression of TGF-β in the aortic tissues of AD patients in comparison to that of patients with coronary artery disease (CAD) receiving coronary artery bypass grafting.

## METHODS

Twenty consecutive patients with AD receiving an ascending aorta replacement in this hospital were included in the study group (AD group). Twenty patients with CAD referred to this hospital for coronary artery bypass grafting were taken as controls (CAD group). Totally, 25 patients (including all 20 patients from the AD group and 5 patients from the CAD group) were operated on an urgent basis, and 15 patients of the CAD group received an elective operation. The patients' information is shown in Table 1.

**Table 1:** Patients’ demographic data.

Group	Aortic dissection	Coronary artery disease	*P*-value
Case, n	20	20	NS
Gender, male/female, n	17/3	15/5	NS
Age, year	51.4±8.2	58.4±4.6	NS
Smoking, n	15	16	NS
Alcohol, n	8	12	NS
Hypertension, n	17	15	NS
Diabetes mellitus, n	1	10	NS
Renal failure, n	1	2	NS
Operation, n	Aorta replacement, with or without aortic valve replacement (20)	Off-pump coronary artery bypass (12); on-pump coronary artery bypass (8)	___
Survival rate, %	80	100	NS

NS=not significant

Surgically removed aorta specimens from the patients with AD and from the punch holes of the ascending aorta of the patients receiving coronary artery bypass grafting were collected, properly stored, and evaluated quantitatively by Western blotting assay for TGF-β_1_ and TβRI.

Patients' age and gender did not differ between groups. The aortic tissue specimens were collected immediately after they were severed from the aorta in patients with AD. In patients receiving coronary artery bypass grafting, the tiny aortic tissues measuring 0.2~0.4 cm were taken when the anterior wall of the ascending aorta was punched. The aortic tissues were stored at -80ºC, and then thawed for inspection of TGF-β_1_ and TβRI by Western blotting assay.

### Statistics

Quantitative data were presented as mean ± standard deviation with range and median values. The intergroup differences were compared by independent samples t-test. Linear correlations of the relative gray scales between the two groups were assessed. A two-tailed *P*-value <0.05 was considered statistically significant.

### Ethics

This study conforms to the Declaration of Helsinki and it was approved by the institutional research Ethics Committee board. Informed consent was obtained from each patient.

## RESULTS

### TGF-β_1_ and TβRI Expressions

**Fig. 1:**
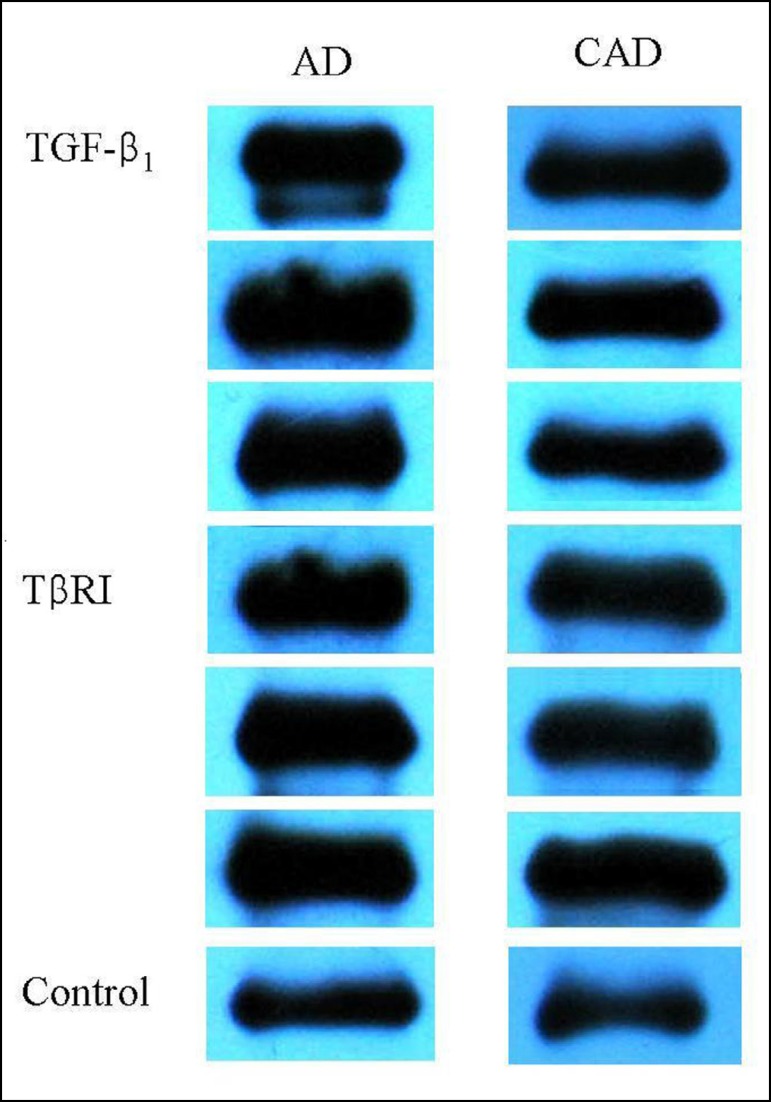
Expressions of transforming growth factor-beta (TGF-β_1_) and transforming growth factor-beta receptor I (TβRI) in the aortic tissues of aortic dissection (AD) and coronary artery disease (CAD) patients as investigated by Western blotting assay.

**Fig. 2:**
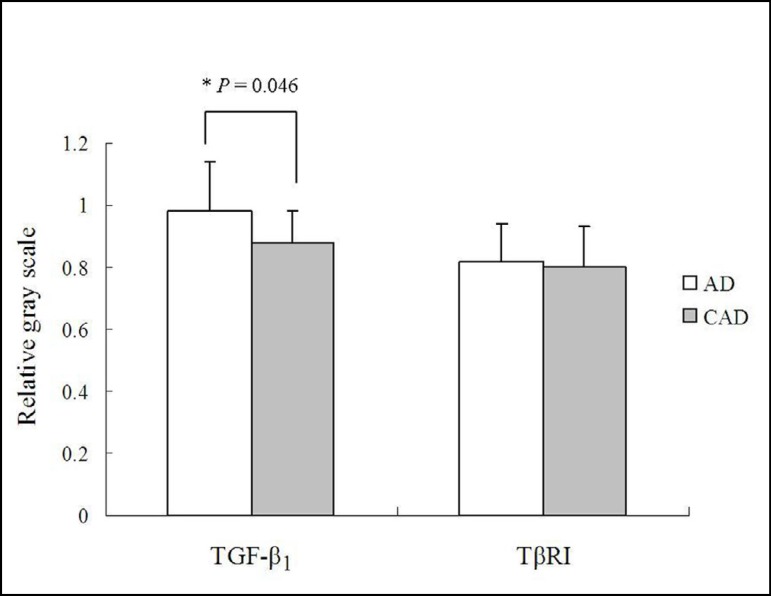
A comparison between the quantitative relative gray scales of transforming growth factor-beta (TGF-β_1_) and transforming growth factor-beta receptor I (TβRI) of the aortic dissection (AD) and the coronary artery disease (CAD) groups. *P=0.046.

TGF-β_1_ and TβRI were positively expressed in the aortic tissues of patients of both AD and CAD groups (Figure 1). The TGF-β_1_ expression in the aortic tissue of AD patients was significantly higher than that of CAD patients. The relative gray scale of the TβRI expression was much higher in AD patients than in CAD patients; however, no significant difference was reached (Figure 2).

### Linear Correlations

**Fig. 3:**
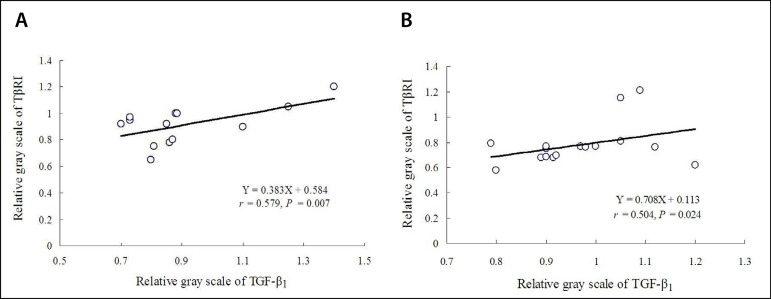
Correlations between the relative gray scales by Western blotting assay: (A) a direct correlation between transforming growth factorbeta (TGF-β_1_) and transforming growth factor-beta receptor I (TβRI) in the aortic dissection group; and (B) a significant direct correlation between TGF-β_1_ and TβRI in the coronary artery disease group.

There was a direct correlation between TGF-β_1_ and TβRI expressions in the AD group (Y=0.383X + 0.584; r²=0.335, r=0.579, *P*=0.007); and there was a significant direct correlation between TGF-β_1_ and TβRI expressions in the CAD group (Y=0.708X + 0.113; r²=0.254, r=0.504, *P*=0.024) (Figure 3). The coefficient was stronger in AD group than in CAD group.

## DISCUSSION

The present study revealed that TGF-β_1_ and TβRI were positively expressed in the aortic tissues of AD and CAD patients, with a direct correlationship between TGF-β_1_ and TβRI expressions in patients of AD and CAD groups. The results illustrated that the aortic wall remodeling happened in response to atherosclerotic or dissecting insults, and the close relationships between them revealed the mutual biological effects between the ligands and the receptors. The disparity of the biomarkers' expressions between the two groups warranted another approach for the understanding of possible differences in aortic remodeling fashions between groups in terms of nature, mechanism, mode, and activities of the TGF signaling.

The expressions of cytokines of the TGF-β signaling pathway depend on the E3 ubiquitin-protein ligases Smurf1 and Smurf2. Knockdown of endogenous Smurf1 or Smurf2 by ribonucleic acid (RNA) interference significantly suppressed the anti-inflammatory effects of TGF-β_1_^[11]^. TGF-β is a potent regulator of vascular development and remodeling, contributing to atherosclerotic process and restenosis by augmenting neointimal proliferation and collagen accumulation^[12]^. TGF-β could activate the phosphatidylinositol-3-kinase (PI3K)/protein kinase B (Akt) pathway in normal murine mammary gland (NmuMG) epithelial cells 30 minutes after treatment, and the activation reached a peak value at 2 hours^[13]^. It could enhance the activities of cyclin dependent kinase inhibitors, p15Ink4B and p21Waf1/Cip1^[14,15]^, thereby influencing the sensitivity of cell proliferation and apoptosis. TGF-β activates p38 mitogen-activated protein kinase (MAPK), which is essential for TGF-β-induced apoptosis and for epithelial-to-mesenchymal transition (EMT)^[16]^.

TGF-β induces extracellular matrix synthesis and prevents enzymatic breakdown of the extracellular matrix by activating Smad and upregulating the expressions of fibronectin and connective tissue growth factor, etc.^[17]^. Other pro-fibrotic factors, such as angiotensin II and advanced glycation end products, might also activate the Smad signaling pathway via TGF-β-dependent or independent mechanisms^[18]^. The Smads show diverse functions based on different family members. In vascular smooth muscle cells, the overexpression of Smad7 inhibits the expressions of fibronectin and connective tissue growth factor, whereas Smad2/3 and Smad4 are for deposition of the extracellular matrix mediated by TGF-β. Alternatively, Smad2/3 signaling pathway could be activated in a TGF-β dependent manner 24 hours after stimulating vascular smooth muscle cells by angiotensin II^[19]^.

TGF-β has been identified as a key anti-atherogenic agent due to its fibrosis-stimulating effects. TβRI is usually expressed in unstable plaques, whereas TβRII is expressed in normal vessels. A study showed that TβRI increased by two folds 8 hours after vascular injury, TGF-β_1_ increased by ten folds within 24 hours, and TβRII increased by three folds^[20]^. Experimental atherosclerosis was observed to be accelerated when TGF-β_1_ was suppressed^[21]^. However, in advanced atherosclerosis, TGF-β_1_ may behave as a proatherogenic stimulus by increasing extracellular matrix formation and fibrosis^[22]^. In such occasions, TGF-β_1_ was likely to lose its anti-atherogenic effects^[23,24]^. Thus, in humans with atherosclerosis, decreased TGF-β_1_ signaling and loss of p27 expression could be found^[25]^. Attenuated TGF-β_1_ signaling and expressions might be an attribution of vascular aging caused by atherosclerosis^[26]^.

Enhanced TGF-β signaling transduction and weakened receptor kinase were found in patients with thoracic aortic aneurysm^[27]^. Jones et al.^[27]^ discovered TGF-β signaling transduction dysfunction in the mouse model of thoracic aorta aneurysm, and they revealed the inherent close relationships between the expression of TGF-β/Smads and the phenotype of vascular smooth muscle cells. TGF-β/Smads play a role in molecular regulation in the reversion of vascular smooth muscle cells, from a synthetic phenotype to a differentiated phenotype^[28]^. An experiment on injured carotid arterial smooth muscle cells of Sprague-Dawley rats with the use of reverse transcription-polymerase chain reaction and Western blotting assays showed that TGF-β_1_ stimulated smooth muscle cell proliferations in a concentration-dependent manner. The expressions of messenger ribonucleic acid (mRNA) and proteins of TGF-β_1_ and TβRII were much higher in the injured carotid artery than in the control group. However, no difference was found in the expressions of TβRI mRNA and protein between the two groups. It illustrated that after vascular injury, the phenotype of the smooth muscle cells changed with unusual proliferations, leading to increased synthesis and secretion of TGF-β_1_. The results verified the functions of TGF-β_1_ in promoting proliferation of the injured smooth muscle cells depending on various receptor subtypes^[29]^.

It has been reported that TGF-β_1_ was unevenly distributed in the aortic wall of patients with AD and of heart donors: it was expressed the highest in the media, followed by a higher expression in the intima. Intergroup comparisons revealed that TGF-β_1_ expression in the entire aortic wall, media, and adventitia were much higher in patients with AD than in heart donor control subjects^[30]^. Transgenic mouse models of Marfan syndrome also disclosed a key role of increased TGF-β signaling in promoting vascular remodeling, dilation, and aneurysmal formation^[31]^. Therefore, TGF-β plays a protective role in controlling excessive activations of monocytes and macrophages, inhibiting matrix degradation, and promoting survival of the smooth muscle cells of the aortic media^[32]^.

When TGF-β/Smad signaling pathway is interfered with TβRI, angiogenesis and production of matrix metalloproteinases may be reduced, whereas the non-Smad dependent pathway depends on TβRI, and may enhance the fibrinolytic function of the extracellular matrix^[27]^. It was demonstrated that TGF-β may have anti-inflammatory and fibrosis-promoting effects, and may also prevent unstable plaque rupture^[33]^. The inhibitory effect of TβRII to TGF-β may promote fibrosis and reduce tissue inflammation^[34]^. By using neutralizing antibodies of TGF-β_1_, β_2_, and β_3_ to block the signaling transduction, there would be an association with an accelerated atherosclerosis and an unstable plaque phenotype^[35]^. Although TGF-β_1_ can inhibit proliferation, it may enhance early immigration of the injured tissue in the mesenchymal cells by increasing the production of matrix metalloproteinase-1^[36]^. Animal experiments on Marfan models showed that activation of TGF-β and the concurrent upregulation of matrix metalloproteinases seemed to contribute to aortic aneurysm formation^[37]^. Pathologically, AD is typically characterized by medial degeneration, smooth muscle cell depletion, and extracellular matrix degradation^[38]^.

## CONCLUSION

The differential expressions of cytokines of TGF-β signaling pathways supported the hypotheses of diverse vascular remodeling fashions between AD and CAD. The aortic remodeling of AD might differ from that of CAD concerning the nature, mechanism, mode, and activities of TGF signaling pathway. The vascular remodeling might be a degradation of the extracellular matrix by the upregulation of matrix metalloproteinases in the aorta of AD patients, while it might be a deposition of the extracellular matrix in the aorta of CAD patients. The development of AD could be associated with a significantly enhanced function of TGF-β/Smad signaling transduction as a result of aortic remodeling incorporating both vascular injury and repair.

**Table:** 

Authors’ roles & responsibilities	
SMY	Substantial contributions to the conception or design of the work; or the acquisition, analysis, or interpretation of data for the work; final approval of the version to be published
HL	Substantial contributions to the conception or design of the work; or the acquisition, analysis, or interpretation of data for the work; final approval of the version to be published
